# Supplementary material for: Short-term social isolation acts on hypothalamic neurons to promote social behavior in a sex- and context-dependent manner
Source: eLife. 2025 Mar 4;13:RP94924. doi: 10.7554/eLife.94924 (PMC11879111; doi:10.7554/eLife.94924)
Supplement: Supplementary file 1. [file elife-94924-supp1.docx]

| **Figure, comparison, and statistical test** | **Group means +/- SD** | **Test results** |
| --- | --- | --- |
| Fig. 1B: total time spent in resident-initiated investigation   - T-test | Group-housed residents: 127.8 ± 88.19 (N = 12)  Single-housed residents: 429.2 ± 161.3 (N =13) | t(23) = -5.72, **P < 0.001** |
| Fig. 1C: proportion of subject females that mounted   - Z-test for independent proportions | Group-housed residents = 0 of 12  Single-housed residents = 11 of 13 | Z = -4.26, **P < 0.001** |
| Fig. 1D: total USVs   - Mann-Whitney U test | Pairs with group-housed resident: 64.67 ± 132.0  (N = 12)  Pairs with single-housed resident: 2042 ± 1292  (N = 13) | Z = 4.16, **P < 0.001** |
| Fig. 1F, left: total number of Fos-positive cells in the POA   - Two-way ANOVA (factor 1 = housing; factor 2 = social test); post-hoc Tukey’s HSD tests | Group-housed baseline: 272.1 ± 63.3 (N = 8)  Group-housed social: 342.2 ± 88.7 (N = 12)  Single-housed baseline: 245.9 ± 38.7 (N = 8)  Single-housed social: 484.9 ± 139.2 (N = 13) | Main effect of housing: F(1,37) = 3.43, P = 0.07  Main effect of social test: F(1,37) = 24.15, **P < 0.01**  Interaction F(1,37) = 7.22, **P = 0.01**  Post-hoc pairwise comparisons:   - Group-housed baseline vs. group-housed social: t(37) = -1.56, P = 0.41 - Group-housed baseline vs. single-housed baseline: t(37) = 0.54, P = 0.95 - Group-housed baseline vs. single-housed social: t(37) = -4.82, **P < 0.001** - Group-housed social vs. single-housed baseline: t(37) = -2.15, P = 0.16 - Group-housed social vs. single-housed social: t(37) = -3.63, **P = 0.005** - Single-housed baseline vs. single-housed social: t(37) = -5.42, **P < 0.001** |
| Fig. 1F, middle: total number of Fos-positive cells in the VMH   - Two-way ANOVA (factor 1 = housing; factor 2 = social test) | Group-housed baseline: 86.5 ± 19.8 (N = 8)  Group-housed social:  98.0 ± 33.2 (N = 12)  Single-housed baseline: 76.8 ± 17.9 (N = 8)  Single-housed social:  98.3 ± 28.9 (N = 13) | Main effect of housing: F(1,37) = 0.29, P = 0.59  Main effect of social test: F(1,37) = 3.63, P = 0.06  Interaction F(1,37) = 0.33, P = 0.56 |
| Fig. 1F, right: total number of Fos-positive cells in the PAG   - Two-way ANOVA (factor 1 = housing; factor 2 = social test); post-hoc Tukey’s HSD tests | Group-housed baseline: 94.9 ± 19.3 (N = 8)  Group-housed social: 95.18 ± 33.12 (N = 11)  Single-housed baseline: 85.1 ± 29.2 (N = 8)  Single-housed social: 145.8 ± 28.4 (N = 13) | Main effect of housing: F(1,36) = 4.93, **P = 0.03**  Main effect of social test: F(1,36) = 10.97, **P < 0.01**  Interaction F(1,36) = 10.75, **P < 0.01**  Post-hoc pairwise comparisons:   - Group-housed baseline vs. group-housed social: t(36) = -0.02, P = 1.00 - Group-housed baseline vs. single-housed baseline: t(36) = 0.95, P = 0.90 - Group-housed baseline vs. single-housed social: t(36) = -3.98, **P = 0.002** - Group-housed social vs. single-housed baseline: t(36) = -0.76, P = 0.87 - Group-housed social vs. single-housed social: t(36) = -4.34, **P < 0.001** - Single-housed baseline vs. single-housed social: t(36) = -4.74, **P < 0.001** |
| Fig. 1G, top: effect of re-group-housing on total resident-initiated investigation   - Friedman test; post-hoc pairwise Wilcox tests with Bonferroni-corrected p values | Re-grouped, day 0:  103.5 ± 120.0 (N = 6)  Re-grouped, day 3:  450.0 ± 228.4 (N = 6)  Re-grouped, day 17:  158.3 ± 67.6 (N = 6) | Main effect of time: X^2(2) = 7, **P = 0.03**  Post-hoc pairwise comparisons:   - Day 0 vs. Day 3: p = 0.03 - Day 0 vs. Day 17: p = 0.28 - Day 3 vs. Day 17: p = 0.08 |
| Fig. 1G, bottom: effect of 17-days isolation on total resident-initiated investigation   - One-way ANOVA with repeated measures; post-hoc Tukey’s HSD tests | 17-day-single, day 0:  103.5 ± 54.9 (N = 6)  17-day-single, day 3:  543.3 ± 291.9 (N = 6)  17-day-single, day 17:  648.8 ± 241.0 (N = 6) | Main effect of time: F(2,5) = 8.65, **P = 0.02**  Post-hoc pairwise comparisons:   - Day 0 vs. Day 3: t(5) = -3.35, **P = 0.04** - Day 0 vs. Day 17: t(5) = -5.79, **P = 0.005** - Day 3 vs. Day 17: t(5) = 0.59, P = 0.83 |
| Fig. 1H, top: effect of re-group-housing on total resident-initiated mounting time   - Friedman test; post-hoc Wilcoxon exact tests with Bonferroni-corrected p values | Re-grouped, day 0:  0.17 ± 0.41 (N = 6)  Re-grouped, day 3:  6.67 ± 7.45 (N = 6)  Re-grouped, day 17:  0.83 ± 1.33 (N = 6) | Main effect of time: X^2(2) = 11.99, P = 0.03  Bonferroni-corrected p value = 0.0167  Post-hoc pairwise comparisons:   - Day 0 vs. Day 3: W = 3.5, **p = 0.015** - Day 0 vs. Day 17: W = 14, p = 0.45 - Day 3 vs. Day 17: W = 30, p = 0.08 |
| Fig. 1H, bottom: effect of 17-days isolation on total resident-initiated mounting   - Friedman test | 17-day-single, day 0:  0.0 ± 0.0 (N = 6)  17-day-single, day 3: 1.67 ± 2.07 (N = 6)  17-day-single, day 17:  1.5 ± 1.97 (N = 6) | X^2(2) = 5.81, P = 0.32 |
| Fig. 1I, top: effect of re-group-housing on total USVs   - One-way ANOVA with repeated measures; post-hoc Tukey’s HSD tests | Re-grouped, day 0:  179.8 ± 190.2 (N = 6)  Re-grouped, day 3:  2207.2 ± 1486.9 (N = 6)  Re-grouped, day 17:  371.5 ± 235.9 (N = 6) | Main effect of time: F(2,5) = 10.41, **P = 0.02**  Post-hoc pairwise comparisons:   - Day 0 vs. Day 3: t(5) = -3.35, **P = 0.04** - Day 0 vs. Day 17: t(5) = -1.95, P = 0.22 - Day 3 vs. Day 17: t(5) = -3.11, P = 0.06 |
| Fig. 1I, bottom: effect of 17-days isolation on total USVs   - One-way ANOVA with repeated measures; post-hoc Tukey’s HSD tests | 17-day-single, day 0:  177.3 ± 180.2 (N = 6)  17-day-single, day 3: 2089.2 ± 1204.8 (N = 6)  17-day-single, day 17:  2881.2 ± 1694.3 (N = 6) | Main effect of time: F(2,5) = 16.04, **P = 0.006**  Post-hoc pairwise comparisons:   - Day 0 vs. Day 3: t(5) = -4.19, **P = 0.02** - Day 0 vs. Day 17: t(5) = -4.16, **P = 0.02** - Day 3 vs. Day 17: t(5) = 2.60, P = 0.10 |
| Fig. 1J: Total Fos-positive POA cells   - T-test | Single:  469.9 ± 45.1 (N = 6)  Re-grouped:  328.4 ± 39.66 (N = 6) | t(10) = 5.77, **P < 0.001** |
| Fig. 1- figure supplement 1C, left: total resident-initiated investigation vs. total Fos-positive POA neurons   - Linear regression | See above for group means and standard deviations | Group-housed residents:   - R^2 = 0.47, t(10) = 2.98, **p = 0.01**   Single-housed residents:   - R^2 = 0.32, t(11) = 2.31, **p = 0.04** |
| Fig. 1- figure supplement 1C, middle: total resident-initiated mounting vs. total Fos-positive POA neurons   - Linear regression; single-housed only | See above for group means and standard deviations | Single-housed residents:   - R^2 = 0.42, t(11) = 2.94, **p = 0.01** |
| Fig. 1- figure supplement 1C, right: total USVs vs. total Fos-positive POA neurons   - Linear regression | See above for group means and standard deviations | For pairs containing group-housed residents:   - R^2 = 0.32, t(10) = 2.21, p = 0.05   For pairs containing single-housed residents:   - R^2 = 0.23, t(11) = 1.85, p = 0.09 |
| Fig. 1- figure supplement 1D, left: total resident-initiated investigation vs. total Fos-positive VMH neurons   - Linear regression | See above for group means and standard deviations | Group-housed residents:   - R^2 = 0.44, t(10) = 2.82, **p = 0.01**   Single-housed residents:   - R^2 = 0.01, t(11) = -0.42, p = 0.68 |
| Fig. 1- figure supplement 1D, middle: total resident-initiated mounting vs. total Fos-positive VMH neurons   - Linear regression; single-housed only | See above for group means and standard deviations | Single-housed residents:   - R^2 < 0.01, t(11) = -0.32, p = 0.75 |
| Fig. 1- figure supplement 1D, right: total USVs vs. total Fos-positive VMH neurons   - Linear regression | See above for group means and standard deviations | For pairs containing group-housed residents:   - R^2 = 0.34, t(10) = 2.28, **p = 0.04**   For pairs containing single-housed residents:   - R^2 = 0.01, t(11) = -0.34, p = 0.74 |
| Fig. 1- figure supplement 1E, left: total resident-initiated investigation vs. total Fos-positive PAG neurons   - Linear regression | See above for group means and standard deviations | Group-housed residents:   - R^2 = 0.006, t(9) = -0.23, p = 0.82   Single-housed residents:   - R^2 = 0.05, t(11) 0.78, p = 0.45 |
| Fig. 1- figure supplement 1E, middle: total resident-initiated mounting vs. total Fos-positive PAG neurons   - Linear regression; single-housed only | See above for group means and standard deviations | Single-housed residents:   - R^2 = 0.04, t(11) = 0.72, p = 0.48 |
| Fig. 1- figure supplement 1E, right: total USVs vs. total Fos-positive VMH neurons   - Linear regression | See above for group means and standard deviations | For pairs containing group-housed residents:   - R^2 = 0.01, t(9) = -0.37, p = 0.71   For pairs containing single-housed residents:   - R^2 = 0.04, t(11) = 0.74, p = 0.47 |
| Fig. 2B: effects of chemogenetic inhibition of hypothalamic neurons on resident-initiated investigation   - Two-way ANOVA with repeated measures on one factor (between-subjects factor = group, within-subjects factor = drug); post-hoc Tukey’s HSD tests | POA-social-hM4Di, saline:  661.0 ± 233.5 (N = 17)  POA-social-hM4Di, CNO:  437.10 ± 242.9 (N = 17)  POA-social-GFP, saline: 581.5 ± 201.4 (N = 14)  POA-social-GFP, CNO:  560.4 ± 180 (N = 14)  AH-TRAPed-hM4Di, saline:  474.1 ± 122.5 (N = 12)  AH-TRAPed-hM4Di, CNO:  488.9 ± 120.3 (N = 12)  VMH-TRAPed-hM4Di, saline: 430.8 ± 137.5  (N = 5)  VMH-TRAPed-hM4Di, CNO: 613.5 ± 115.1 (N = 5) | Main effect of group: F(3,44) = 0.85, P = 0.47  Main effect of drug: F(1,44) = 0.05, P = 0.83  Interaction: F(3,44) = 6.73, **P < 0.001**  Within group post-hoc pairwise comparisons:   - POA-social-hM4Di, saline vs. CNO: t(44) = -4.43, **P = 0.0015** - POA-social-GFP, saline vs. CNO: t(44) = -0.38, P = 1.00 - AH-TRAPed-hM4Di, saline vs. CNO: t(44) = 0.56, P = 1.00 - VMH-TRAPed-hM4Di, saline vs. CNO: t(44) = 1.96, P = 0.52   Across group post-hoc pairwise comparisons (saline vs. saline or CNO vs. CNO only):   - AH-TRAPed-hM4Di CNO vs. POA-social-GFP CNO: t(44) = -0.93, P = 0.98 - AH-TRAPed-hM4Di CNO vs. POA-social-hM4Di CNO: t(44) = 0.76, P = 0.99 - AH-TRAPed-hM4Di CNO vs. VMH-TRAPed-hM4Di CNO: t(44) = -1.21, P = 0.92 - POA-social-GFP CNO vs. POA-social-hM4Di CNO: t(44) = 1.81, P = 0.62 - POA-social-GFP CNO vs. VMH-TRAPed-hM4Di CNO: t(44) = -0.54, P = 1.00 - POA-social-hM4Di CNO vs. VMH-TRAPed-hM4Di CNO: t(44) = -1.84, P = 0.60 - AH-TRAPed-hM4Di saline vs. POA-social-GFP saline: t(44) = -1.62, P = 0.74 - AH-TRAPed-hM4Di saline vs. POA-social-hM4Di saline: t(44) = -2.77, P = 0.12 - AH-TRAPed-hM4Di saline vs. VMH-TRAPed-hM4Di saline: t(44) = 0.26, P = 1.00 - POA-social-GFP saline vs. POA-social-hM4Di saline: t(44) = -1.13, P = 0.95 - POA-social-GFP saline vs. VMH-TRAPed-hM4Di saline: t(44) = 1.49, P = 0.81 - POA-social-hM4Di saline vs. VMH-TRAPed-hM4Di saline: t(44) = 2.33, P = 0.30 |
| Fig. 2C: effects of chemogenetic inhibition of hypothalamic neurons on proportion of trials with resident-initiated mounting   - McNemar’s test for paired proportions | POA-social-hM4Di:  7 of 17 on saline day,  1 of 17 on CNO day  POA-social-GFP:  7 of 14 on saline day,  7 of 14 on CNO day  AH-TRAPed-hM4Di:  6 of 12 on saline day,  3 of 12 on CNO day  VMH-TRAPed-hM4Di:  4 of 5 on saline day,  4 of 5 on CNO day | POA-social-hM4Di, CNO vs. saline: X^2 (1) = 4.17, p = **0.04**  POA-social-GFP, CNO vs. saline: X^2 (1) = 0.25, p = 0.62  AH-TRAPed-hM4Di, CNO vs. saline: X^2 (1) = 0.57, p = 0.45  VMH-TRAPed-hM4Di, CNO vs. saline: X^2 (1) = 0, p = 1.00 |
| Fig. 2D: effects of chemogenetic inhibition of hypothalamic neurons on total USVs   - Two-way ANOVA with repeated measures on one factor (between-subjects factor = group, within-subjects factor = drug); post-hoc Tukey’s HSD tests | POA-social-hM4Di, saline:  2756 ± 802.7 (N = 17)  POA-social-hM4Di, CNO:  1250 ± 1080.9 (N =17)  POA-social-GFP, saline: 2221 ± 1034 (N =14)  POA-social-GFP, CNO:  2225 ± 1110 (N =14)  AH-TRAPed-hM4Di, saline:  1694 ± 638.3 (N = 12)  AH-TRAPed-hM4Di, CNO:  1707 ± 783.5 (N =12)  VMH-TRAPed-hM4Di, saline: 1640 ± 771.8 (N =5)  VMH-TRAPed-hM4Di, CNO: 2180 ± 599.6 (N =5) | Main effect of group: F(3,44) = 0.95, P = 0.42  Main effect of drug: F(1,44) = 2.58, P = 0.11  Interaction: F(3,44) = 11.59, **P < 0.001**  Within group post-hoc pairwise comparisons:   - POA-social-hM4Di, saline vs. CNO: t(44) = -6.77, **P < 0.001** - POA-social-GFP, saline vs. CNO: t(44) = 0.017, P = 1.00 - AH-TRAPed-hM4Di, saline vs. CNO: t(44) = 0.048, P = 1.00 - VMH-TRAPed-hM4Di, saline vs. CNO: t(44) = 1.32, P = 0.89   Across group post-hoc pairwise comparisons (saline vs. saline or CNO vs. CNO only):   - AH-TRAPed-hM4Di CNO vs. POA-social-GFP CNO: t(44) = -1.33, P = 0.88 - AH-TRAPed-hM4Di CNO vs. POA-social-hM4Di CNO: t(44) = 1.23, P = 0.92 - AH-TRAPed-hM4Di CNO vs. VMH-TRAPed-hM4Di CNO: t(44) = -0.90, P = 0.98 - POA-social-GFP CNO vs. POA-social-hM4Di CNO: t(44) = 2.74, P = 0.14 - POA-social-GFP CNO vs. VMH-TRAPed-hM4Di CNO: t(44) = 0.09, P = 1.00 - POA-social-hM4Di CNO vs. VMH-TRAPed-hM4Di CNO: t(44) = -1.85, P = 0.59 - AH-TRAPed-hM4Di saline vs. POA-social-GFP saline: t(44) = -1.59, P = 0.75 - AH-TRAPed-hM4Di saline vs. POA-social-hM4Di saline: t(44) = -3.35, **P = 0.03** - AH-TRAPed-hM4Di saline vs. VMH-TRAPed-hM4Di saline: t(44) = -0.12, P = 1.00 - POA-social-GFP saline vs. POA-social-hM4Di saline: t(44) = -1.77, P = 0.65 - POA-social-GFP saline vs. VMH-TRAPed-hM4Di saline: t(44) = 1.33, P = 0.89 - POA-social-hM4Di saline vs. VMH-TRAPed-hM4Di saline: t(44) = 2.61, P = 0.18 |
| Fig. 2E: total movement of POA-social-hM4Di females   - Paired t-test | POA-social-hM4DI, saline:  12509 ± 2550 (N = 17)  POA-social-hM4Di, CNO:  13257 ± 3503 (N = 17) | t(16) = -1.01, P = 0.33 |
| Fig. 2- figure supplement 1A: total TRAPing session resident-initiated investigation vs. change in resident-initiated investigation (CNO-saline) in test sessions   - Linear regression | See above for group means and standard deviations | R^2 = 0.11, t(14) = -1.32, p = 0.21 |
| Fig. 2- figure supplement 1B: total TRAPing session resident-initiated mounting vs. change in resident-initiated mounting (CNO-saline) in test sessions   - Linear regression | See above for group means and standard deviations | R^2 = 0.13, t(14) = -1.45, p = 0.17 |
| Fig. 2- figure supplement 1C: total TRAPing session USVs vs. change in USVs (CNO-saline) in test sessions   - Linear regression | See above for group means and standard deviations | R^2 = 0.11, t(15) = -1.45, p = 0.19 |
| Fig. 2- figure supplement 1E: effects of chemogenetic inhibition of POA neurons on resident-initiated investigation, non-social control females   - Paired t-test | POA-non-social-hM4Di, saline:  307.4 ± 229.4 (N = 5)  POA-non-social-hM4Di, CNO:  256.0 ± 272.9 (N =5) | T(4) = 0.38, p = 0.72 |
| Fig. 2- figure supplement 1F: effects of chemogenetic inhibition of POA neurons on proportion of trials with resident-initiated mounting, non-social control females   - McNemar’s test for paired proportions | Saline: 1 of 5    CNO: 1 of 5 | CNO vs. saline: X^2 (1) = 0, p = 1.00 |
| Fig. 2- figure supplement 1G: effects of chemogenetic inhibition of POA neurons on total USVs, non-social control females   - Paired t-test | POA-non-social-hM4Di, saline:  1442 ± 1375 (N = 5)  POA-non-social-hM4Di, CNO:  1603 ± 879.0 (N = 5) | T(4) = -0.2, p = 0.85 |
| Fig. 3B: effects of caspase-mediated ablation of POA neurons on resident-initiated investigation   - Two-way ANOVA with repeated measures on one factor (between-subjects factor = group, within-subjects factor = time) | POA-social-caspase, pre-4-OHT:  489.0 ± 119.0 (N = 15)    POA-social-caspase, post-4-OHT: 688.8 ± 215.1 (N = 15)    POA-social-GFP, pre-4-OHT:  476.3 ± 155.9 (N = 13)    POA-social-GFP, post-4-OHT: 545.4 ± 155.4 (N = 13) | Main effect of group: F(1,26) = 2.84, P = 0.10  Main effect of time: F(1,26) =10.08, **P = 0.004**  Interaction: F(1,26) = 2.38, P = 0.14 |
| Fig. 3C: effects of caspase-mediated ablation of POA neurons on proportion of trials with resident-initiated mounting   - McNemar’s test for paired proportions | POA-social-caspase:  13 of 15 on saline day,  4 of 15 on CNO day  POA-social-GFP:  8 of 13 on saline day,  6 of 13 on CNO day | POA-social-caspase, CNO vs. saline: X^2 (1) = 4.92, p = **0.03**  POA-social-GFP, CNO vs. saline: X^2 (1) = 0.50, p = 0.48 |
| Fig. 3D: effects of caspase-mediated ablation of POA neurons on total USVs   - Two-way ANOVA with repeated measures on one factor (between-subjects factor = group, within-subjects factor = time); post-hoc Tukey’s HSD tests | POA-social-caspase, pre-4-OHT:  1411.8 ± 771.6 (N = 15)    POA-social-caspase, post-4-OHT:  2024.3 ± 1325.1 (N =15)    POA-social-GFP, pre-4-OHT:  2562.9 ± 1063.9 (N = 13)    POA-social-GFP, post-4-OHT:  2152.9 ± 1043.3 (N =13) | Main effect of group: F(1,26) = 3.11, P = 0.09  Main effect of time: F(1,26) =0.31, P = 0.58  Interaction: F(1,26) = 7.98, **P < 0.01**    Post-hoc pairwise comparisons:  POA-social-caspase pre vs. POA-social-caspase-post: t(26) = 2.48, P = 0.09  POA-social-caspase pre vs. POA-social-GFP-pre: t(26) = -3.31, **P = 0.01**  POA-social-caspase pre vs. POA-social-GFP-post: t(26) = 1.81, P = 0.29  POA-social-caspase post vs. POA-social-GFP-pre: t(26) = -1.34, P = 0.55  POA-social-caspase post vs. POA-social-GFP-post: t(26) = -0.28, P = 0.99  POA-social-GFP pre vs. POA-social-GFP-post: t(26) = -1.55, P = 0.43 |
| Fig. 3- figure supplement 1A: effects of caspase-mediated ablation of POA neurons on resident-initiated investigation   - Two-way ANOVA with repeated measures on one factor (between-subjects factor = group, within-subjects factor = time) | TRAP2 heterozygous, pre-4-OHT:  465.0 ± 125.3 (N = 11)    TRAP2 heterozygous, post-4-OHT:  711.1 ± 173.8 (N =11)    TRAP2 homozygous, pre-4-OHT:  428.2 ± 195.5 (N = 9)    TRAP2 homozygous, post-4-OHT:  447.8 ± 235.5 (N =9) | Main effect of group: F(1,18) = 4.26, P = 0.054  Main effect of time: F(1,18) =12.06, **P = 0.003**  Interaction: F(1,18) = 8.76, **P < 0.01**    Within group post-hoc pairwise comparisons:  Heterozygous pre vs. heterozygous post: t(18) = 4.80, **P < 0.001**  Homozygous pre vs. homozygous post: t(18) = 0.35, P = 0.99  Across group post-hoc pairwise comparisons:  Heterozygous post vs. homozygous post: t(18) = 2.88, **P = 0.045**  Heterozygous post vs. homozygous pre: t(18) = 3.49, **P = 0.01**  Homozygous post vs. heterozygous pre: t(18) = -0.21, P = 1.00  Heterozygous pre vs. Homozygous pre: t(18) = 0.51, P = 0.95 |
| Fig. 3- figure supplement 1B: effects of caspase-mediated ablation of POA neurons on proportion of trials with resident-initiated mounting, non-social control females   - McNemar’s test for paired proportions | TRAP2 heterozygous:  9 of 11 on saline day,  3 of 11 on CNO day  TRAP2 homozygous:  7 of 9 on saline day,  1 of 9 on CNO day | TRAP2 heterozygous, pre-4-OHT vs. post-4-OHT: X^2 (1) = 2.5, p = 0.11  TRAP2 homozygous, pre-4-OHT vs. post-4-OHT: X^2 (1) = 4.17, **p = 0.04** |
| Fig. 3- figure supplement 1C: effects of caspase-mediated ablation of POA neurons on total USV   - Two-way ANOVA with repeated measures on one factor (between-subjects factor = group, within-subjects factor = time) | TRAP2 heterozygous, pre-4-OHT:  1330 ± 844.0 (N = 11)    TRAP2 heterozygous, post-4-OHT:  2303 ± 1446 (N =11)    TRAP2 homozygous, pre-4-OHT:  1392 ± 526.1 (N = 9)    TRAP2 homozygous, post-4-OHT:  1160 ± 782.2 (N =9) | Main effect of group: F(1,18) = 1.88, P = 0.19  Main effect of time: F(1,18) = 3.28, P = 0.09  Interaction: F(1,18) = 8.70, **P < 0.01**    Within group post-hoc pairwise comparisons:  Heterozygous pre vs. heterozygous post: t(18) = 3.55, **P = 0.01**  Homozygous pre vs. homozygous post: t(18) = -0.77, P = 0.87  Across group post-hoc pairwise comparisons:  Heterozygous post vs. homozygous post: t(18) = 2.12, P = 0.18  Heterozygous post vs. homozygous pre: t(18) = 2.10, P = 0.19  Homozygous post vs. heterozygous pre: t(18) = -0.21, P = 1.00  Heterozygous pre vs. Homozygous pre: t(18) = 0.51, P = 0.95 |
| Fig. 3- figure supplement 1D: comparison of counts of Fos-positive POA neurons in female groups following same-sex interactions:   - One-way ANOVA; post-hoc Tukey’s HSD tests | TRAP2 -/- POA-social caspase:  196.4 ± 11.9 (N =5)  Group-housed baseline: 272.1 ± 63.3 (N = 8)  Group-housed social:  342.2 ± 88.7 (N = 12)  Single-housed baseline: 245.9 ± 38.7 (N = 8)  Single-housed social:  484.9 ± 139.2 (N = 13) | Main effect of group: F(4,41) = 13.88, **P < 0.001**  Post-hoc comparisons:  Caspase vs. GH baseline: t(41) = -1.42, P = 0.62  Caspase vs. GH social: t(41) = 2.93, **P = 0.04**  Caspase vs. SH social: t(41) = -0.93, P = 0.88  Caspase vs. SH social: t(41) = -5.87, **P < 0.0001**  GH baseline vs. GH social: t(41) = -1.64, P = 0.48  GH baseline vs. SH baseline: t(41) = -0.56, P = 0.98  GH baseline vs. SH social: t(41) = -5.07, **P = 0.0001**  GH social vs. SH baseline: t(41) = 2.26, P = 0.18  GH social vs. SH social: t(41) = -3.82, **P = 0.004**  SH baseline vs. SH social: t(41) = -5.70, **P < 0.0001** |
| Fig. 3- figure supplement 1E: total resident-initiated investigation vs. total Fos-positive POA neurons | See above for group means and standard deviations | R^2 = 0.06, t(3) = -0.46, p = 0.68 |
| Fig. 3- figure supplement 1F: total USVs vs. total Fos-positive POA neurons | See above for group means and standard deviations | R^2 = 0.28, t(3) = -1.09, p = 0.35 |
| Fig. 4B: USVs per second, solo sessions   - Mann Whitney U test performed on the difference in USV rates | POA-social-ChR2, laser – pre-laser:  1.43 ± 2.49 (N = 9)    POA-social-GFP, laser - pre-laser: 0.0 ± 0.0 (N =6) | Z = 1.71, P = 0.09 |
| Fig. 4C: USVs per second, social sessions   - Mann Whitney U test performed on the difference in USV rates | POA-social-ChR2, laser - pre-laser:  1.98 ± 1.74 (N = 9)    POA-social-GFP, laser - pre-laser:  0.04 ± 0.09 (N = 6) | Z = 2.77, **P = 0.006** |
| In text, related to Fig. 4C: USVs per second, laser vs. pre-laser, according to distance between females at time of optogenetic activation   - Paired t-test | “Near” stimulations for POA-iso-ChR2 mice, laser – pre-laser:  2.97 ± 1.32 (N = 7)    “Far” stimulations for POA-iso-ChR2 mice, laser – pre-laser: 1.84 ± 1.75 (N = 7)    N = 2 females excluded that did not have “far” stimulations | t(6) = 3.07, **P = 0.02** |
| Fig. 4E: percentage of laser stimulations followed by social investigation   - T-test | POA-social-ChR2:  41.5 ± 31.2 (N = 9)  POA-social-GFP:  10.8 ± 5.0 (N = 6) | T(13) = 2.36, **p = 0.03** |
| Fig. 4G: mean duration of social investigation bout, comparing bouts overlapping with periods of laser stimulation vs. bouts non-overlapping   - Two-way ANOVA with repeated measures on one factor (between-subjects factor = group, within-subjects factor = laser overlap) | POA-social-ChR2, overlapping with laser:  5.75 ± 2.37 (N = 8)  POA-social-ChR2, non-overlapping with laser:  2.07 ± 0.88 (N = 8)  POA-social-GFP, overlapping with laser:  3.95 ± 2.14 (N = 6)  POA-social-GFP, non-overlapping with laser:  2.72 ± 0.75 (N = 6)  N = 1 ChR2 female excluded that did not have any social investigation bouts overlapping with periods of laser stimulation | Main effect of group: F(1,12) = 0.60, P = 0.45  Main effect of laser: F(1,12) =19.97, **P < 0.003**  Interaction: F(1,12) = 4.96, **P = 0.046**    Within group post-hoc pairwise comparisons:  POA-social-ChR2 overlapping with laser vs. non-overlapping: t(12) = 5.11, **P = 0.001**  POA-social-GFP overlapping with laser vs. non-overlapping: t(12) = 1.48, P = 0.47  Across group post-hoc pairwise comparisons:  POA-social-ChR2 overlapping vs. POA-social-GFP overlapping: t(12) = 1.47, P = 0.49  POA-social-ChR2 overlapping vs. POA-social-GFP non-overlapping: t(12) = 3.47, **P = 0.02**  POA-social-GFP overlapping vs. POA-social-ChR2 non-overlapping: t(12) = 1.93, P = 0.27  POA-social-ChR2 non-overlapping vs. POA-social-GFP non-overlapping: t(12) = -1.44, P = 0.50 |
| Fig. 5B: effects of chemogenetic inhibition of POA neurons on resident-initiated investigation, females GH during TRAPing   - Paired t-test | Saline:  535.6 ± 203.9 (N = 5)  CNO:  516.0 ± 220.1 (N = 5) | T(4) = 0.15, p = 0.88 |
| Fig. 5D: effects of chemogenetic inhibition of POA neurons on total USVs, females GH during TRAPing   - Paired t-test | Saline:  2130 ± 1552 (N = 5)  CNO:  1654 ± 1048 (N = 5) | T(4)= 1.22, p = 0.29 |
| Fig. 5F, left: proportion of mice with non-zero USV rates, pre-laser vs. laser, solo sessions, females GH during TRAPing   - McNemar’s test for paired proportions | Pre-laser: 0 of 6  During laser: 1 of 6 | X^2(1) = 0, p = 1.00 |
| Fig. 5F, right: proportion of mice with non-zero USV rates, pre-laser vs. laser, social sessions, females GH during TRAPing   - McNemar’s test for paired proportions | Pre-laser: 2 of 6  During laser: 3 of 6 | X^2(1) = 0, p = 1.00 |
| Fig. 5G: percentage of laser stimulations followed by social investigation   - T-test | POA-social-ChR2, female GH during TRAPing:  41.5 ± 31.2 (N = 9)  POA-social-GFP:  10.8 ± 5.0 (N = 6) | t(10) = 0.21, p = 0.84 |
| Fig. 5J: effects of chemogenetic inhibition of POA neurons on resident-initiated investigation, females tested as GH   - Paired t-test | Saline:  38.3 ± 23.8 (N = 7)  CNO:  34.7 ± 25.1 (N = 7) | t(6) = 0.99, p = 0.36 |
| Fig. 5L: effects of chemogenetic inhibition of POA neurons on total USVs, females tested as GH   - Paired t-test | Saline:  137.0 ± 127.1 (N = 7)  CNO:  15.3 ± 14.8 (N = 7) | t(6) = 2.47, **p = 0.048** |
| Fig. 5M: effects of chemogenetic inhibition of POA neurons on resident-initiated investigation, females tested as SH   - Paired t-test | Saline:  389.9 ± 87.7 (N = 7)  CNO:  78.9 ± 29.3 (N = 7) | t(6) = 8.52, **p < 0.001** |
| Fig. 5N: effects of chemogenetic inhibition of POA neuron on resident-initiated mounting time, females tested as SH   - McNemar’s test for paired proportions | Saline: 6 of 7  CNO: 0 of 7 | X^2(1) = 4.167, **p = 0.041** |
| Fig. 5O: effects of chemogenetic inhibition of POA neurons on total USVs, females tested as SH   - Paired t-test | Saline:  2136 ± 565.5(N = 7)  CNO:  45.0 ± 11.0 (N = 7)  Saline:  2136 ± 565.5(N = 7)  CNO:  45.0 ± 11.0 (N = 7) | t(6) = 9.72, **p < 0.0001** |
| Fig. 7A: total resident-initiated social investigation   - Two-way ANOVA (factor 1 = housing; factor 2 = social context) | Male-female, group-housed resident:  291.0 ± 152.2 (N = 8)  Male-female, single-housed resident:  455.1 ± 95.8 (N = 8)  Male-male, group-housed resident:  214.1 ± 73.8 (N = 7)  Male-male, single-housed resident:  246.8 ± 68.5 (N = 7) | Main effect of housing: F(1,26) = 6.55, **P = 0.02**  Main effect of social context: F(1,26) = 13.74, **P < 0.001**  Interaction: F(1,26) = 2.92, P = 0.10 |
| Fig. 7B: proportion of trials with resident-initiated mounting   - Z-test for independent proportions | Male-female, group-housed resident: 2 of 8  Male-female, single-housed resident: 6 of 8  Male-male, group-housed resident: 1 of 7  Male-male, single-housed resident: 0 of 7 | Male-female, group-housed resident vs. single-housed resident:  Z = -2, **P = 0.046**  Male-male, group-housed resident vs. Single-housed resident:  Z = 1.04, P = 0.30 |
| Fig. 7C: total USVs   - Two-way ANOVA (factor 1 = housing; factor 2 = social context); post-hoc Tukey’s HSD tests | Male-female, group-housed resident:  545.3 ± 282.5 (N =8)    Male-female, single-housed resident: 2076.9 ± 462.2 (N = 8)    Male-male, group-housed resident: 8.4 ± 7.9 (N = 7)    Male-male, single-housed resident: 21.1 ± 16.4 (N = 7) | Main effect of housing: F(1,26) = 56.29, **P < 0.001**  Main effect of social context: F(1,26) = 158.64, **P < 0.001**  Interaction: F(1,26) = 54.45, **P < 0.001**    Post-hoc pairwise comparisons   - MF group vs. MF single: t(26) = -10.89, **P < 0.001** - MF group vs. MM group: t(26) = 3.69, **P = 0.006** - MF group vs. MM single: t(26) = 3.60, **P = 0.007** - MF single vs. MM group: t(26) = 14.21, **P < 0.001** - MF single vs. MM single: t(26) = 14.12, **P < 0.001** - MM single vs. MM group: t(26) = -0.09, P = 1.00 |
| Fig. 7D: total Fos-positive POA neurons   - Two-way ANOVA (factor 1 = housing; factor 2 = social context); post-hoc Tukey’s HSD tests | Male-female, group-housed resident: 229.9 ± 68.3 (N = 8)    Male-female, single-housed resident: 328.2 ± 69.5 (N = 8)    Male-male, group-housed resident: 227.0 ± 53.8  (N = 7)    Male-male, single-housed resident: 227.3 ± 42.7  (N = 7) | Main effect of housing: F(1,26) = 4.97, **P = 0.04**  Main effect of social context: F(1,26) = 5.52, **P = 0.03**  Interaction: F(1,26) = 4.91, **P = 0.04**    Post-hoc pairwise comparisons   - MF group vs. MF single: t(26) = -3.25, **P = 0.02** - MF group vs. MM group: t(26) = 0.09, P = 1.00 - MF group vs. MM single: t(26) = 0.09, P = 1.00 - MF single vs. MM group: t(26) = 3.24, **P = 0.02** - MF single vs. MM single: t(26) = 3.23, **P = 0.02** - MM single vs. MM group: t(26) = -0.01, P = 1.00 |
| Fig. 7F: effects of chemogenetic inhibition of male POA-social neurons on resident-initiated investigation   - Two-way ANOVA (factor 1 = group; factor 2 = drug) | Male POA-social-hM4Di, saline:  476.6 ± 88.4 (N = 10)    Male POA-social-hM4Di, CNO:  536.4 ± 208.6 (N = 10)    Male POA-social-GFP, saline:  292.8 ± 109.7 (N = 10)  Male POA-social-GFP, CNO:  325.0 ± 118.6 (N = 10) | Main effect of group: F(1,18) = 13.76, **P = 0.002**  Main effect of drug: F(1,18) = 2.05, P = 0.17  Interaction: F(1,18) = 0.18, P = 0.67 |
| Fig. 7G: effects of chemogenetic inhibition of male POA-social neurons on proportion of trials with resident-initiated mounting   - McNemar’s test for paired proportions | Male POA-social-hM4Di, saline: 6 of 10  Male POA-social-hM4Di, CNO: 0 of 10  Male POA-social-GFP, saline: 9 of 10  Male POA-social-GFP, CNO: 9 of 10 | Male POA-social-hM4Di, CNO vs. saline: X^2 (1) = 4  167, p = **0.04**  Male POA-social-GFP, CNO vs. saline: X^2 (1) = 0  167, p = 1.0 |
| Fig. 7H: effects of chemogenetic inhibition of male POA-social neurons on total USVs   - Two-way ANOVA (factor 1 = group; factor 2 = drug) | Male POA-social-hM4Di, saline:  1868 ± 1097.4 (N = 10)    Male POA-social-hM4Di, CNO:  1826.4 ± 1120.5 (N = 10)    Male POA-social-GFP, saline:  1587.3 ± 764.1 (N = 10)    Male POA-social-GFP, CNO:  1643.9 ± 674.6 (N = 10) | Main effect of group: F(1,18) = 0.35, P = 0.56  Main effect of drug: F(1,18) = 0.00, P = 0.96  Interaction: F(1,18) = 0.12, P = 0.74 |
| Fig. 7- figure supplement 1A: comparison of TRAPing session resident-initiated social investigation for POA-social-hM4Di groups   - T-test | Male POA-social-hM4Di:  365.8 ± 140.0 (N = 8)  Female POA-social-hM4Di:  553.9 ± 194.7 (N = 16) | t(22) = 2.43, **P = 0.02** |
| Fig. 7- figure supplement 1B: comparison of TRAPing session mounting for POA-social-hM4Di groups   - Mann Whitney U test | Male POA-social-hM4Di:  190.5 ± 154.9 (N = 8)  Female POA-social-hM4Di:  23.3 ± 45.6 (N = 16) | Z = -2.88, **P = 0.004** |
| Fig. 7- figure supplement 1C: comparison of TRAPing session USVs for POA-social-hM4Di groups   - T-test | Male POA-social-hM4Di:  1678 ± 1024 (N = 8)  Female POA-social-hM4Di:  2534 ± 637.1 (N = 17) | t(23) = -2.03, P = 0.054 |
